# Leucine-rich repeat containing 4 act as an autophagy inhibitor that restores sensitivity of glioblastoma to temozolomide

**DOI:** 10.1038/s41388-020-1312-6

**Published:** 2020-05-05

**Authors:** Jianbo Feng, Yan Zhang, Xing Ren, Di Li, Haijuan Fu, Changhong Liu, Wen Zhou, Qing Liu, Qiang Liu, Minghua Wu

**Affiliations:** 1 Hunan Provincial Tumor Hospital and the Affiliated Tumor Hospital of Xiangya Medical School, Central South University, Changsha, 410006 Hunan China; 2 Cancer Research Institute, School of Basic Medical Science, Central South University, Changsha, 410078 Hunan China; 3 Key Laboratory of Carcinogenesis and Cancer Invasion, Ministry of Education, Changsha, 410078 Hunan China; 4 Key Laboratory of Carcinogenesis, Ministry of Health, Changsha, 410078 Hunan China; 5 Institute of Medical Sciences, The Second Hospital of Shandong University, Jinan, 250033 Shandong China; 6 Xiangya Hospital, Central South University, Changsha, 410008 Hunan China; 7 Third Xiangya Hospital, Central South University, Changsha, 410013 Hunan China

**Keywords:** CNS cancer, Autophagy

## Abstract

Temozolomide (TMZ) insensitivity and resistance are major causes of treatment failure and poor prognosis for GBM patients. Here, we identify LRRC4 as a novel autophagy inhibitor that restores the sensitivity of GBMs to TMZ. LRRC4 was associated with the DEPTOR/mTOR complex, and this interaction resulted in autophagy inhibition. Further investigation demonstrated that the PDZ binding domain of LRRC4 binds to the PDZ domain of DEPTOR. This binding decreases the half-life of DEPTOR via ubiquitination, thus inhibiting GBM cell autophagy and increasing the TMZ treatment response of GBM. Combined LRRC4 expression and TMZ treatment prolonged the survival of mice with tumour xenografts. Furthermore, the levels of LRRC4, DEPTOR and autophagy are clinically relevant for GBM, indicating that LRRC4 is likely to have significant potential as a therapeutic marker and target for TMZ treatment in glioma patients.

## Introduction

Glioblastoma (GBM) is the most common malignant primary brain tumour in adults and one of the most lethal human cancers [1], with an average survival of slightly over 1 year after the initial diagnosis [2]. Diffuse invasion of tumour cells into surrounding brain tissue is a common characteristic of malignant glioma and is responsible for treatment failure or relapse even when maximal surgical resection is performed [3]. Moreover, GBM is among the most resistant cancer types to radiation and cytotoxic chemotherapy [4]. Although temozolomide (TMZ) has been clinically shown to prolong GBM patient survival, the treatment outcome is frequently dependent on O6-methylguanine-DNA methyltransferase (MGMT) expression. In glioma, patient sensitivity to TMZ and the associated enhanced overall survival are frequently linked to low MGMT expression and increased MGMT promoter methylation [5]. However, the TMZ resistance phenomenon can still occur, and clinical trials that aiming to overcome TMZ resistance by exploring the modulation of MGMT or the addition of targeted agents cannot efficiently improve treatment efficacy [6]. Thus, a deeper understanding of the mechanisms contributing to TMZ resistance could provide prospective molecular targets for glioma therapy.

Autophagy is an evolutionarily conserved catabolic process that involves the sequestration and transport of damaged organelles and misfolded and dysfunctional proteins to the lysosomes for degradation [7]. Following lysosomal degradation, cells restore the intracellular nutrient supply during starvation and protect the cell against damage caused by toxic macromolecules and damaged organelles [8, 9]. In addition to nutrient status and intracellular damage, environmental stressors such as hypoxia [10], ER stress [11] and reactive oxygen species accumulation [12] can also induce autophagy. Normal autophagy plays important physiological roles in human health, and abnormal autophagy leads to various diseases, such as neurodegenerative disorders [13] and cancer progression [14]. The role of autophagy differs in different stages of cancer development; initially, autophagy likely has a preventive effect against cancer, but once a tumour develops, the cancer cells could utilize autophagy for their own cytoprotection [15]. It seems that autophagy plays dual roles in cancer progression. Appropriate autophagy acts as a cytoprotective mechanism leading to tumour cell apoptosis resistance, however, excessive autophagy promotes tumour cell death. Recent studies have emphasized the role of autophagy in tumour promotion [16] and drug resistance [17], and inhibiting autophagy significantly increased chemotherapy efficacy [18]; however, the underlying mechanism connecting autophagy and drug resistance is not clear. It is of paramount importance to elucidate the mechanism of autophagy in chemotherapy resistance.

Leucine-rich repeat containing 4 (LRRC4), also called netrin-G ligand-2, is a member of the leucine-rich repeat (LRR) superfamily [19]. Previous studies have confirmed that LRRC4 plays a central role in early nervous system development and differentiation, especially during synapse formation [20–22]; in addition, our group first confirmed a new function of LRRC4 as a tumour suppressor for glioma [23, 24]. LRRC4 reduced the activity of the Ras/c-Raf/ERK/MAPK signalling pathways and inhibited GBM cell proliferation and invasion [25, 26]. Mechanistically, LRRC4 abolished ERK1/2 activation and inhibited ERK1/2 nuclear translocation through direct interaction with ERK1/2 and then inhibited ERK1/2 binding to MEK [27]. Moreover, our recent study also found that LRRC4 plays an important role in the GBM immunemicroenvironment. LRRC4 bound to phosphoinositide-dependent protein kinase 1 and HSP90 to promote NF-κB translocation and cytokine production in GBM cells and influenced the infiltration of Treg cells in the GBM microenvironment [28].

Here, we first demonstrated that LRRC4 is a novel inhibitor of autophagy. LRRC4 directly interacted with DEPTOR (DEP domain containing mTOR interacting protein), which, as a mTOR inhibitor, bound to mTORC1 and mTORC2 via the PDZ domain. LRRC4 decreased the protein level of DEPTOR, which resulted in mTOR activation, thereby decreasing the cellular autophagy level. LRRC4 inhibited cell autophagy, restoring TMZ treatment sensitivity in GBM, which could be used as a possible therapeutic strategy.

## Results

### LRRC4 is negatively associated with autophagy signals and glioma patient outcomes

**Fig. 1:**
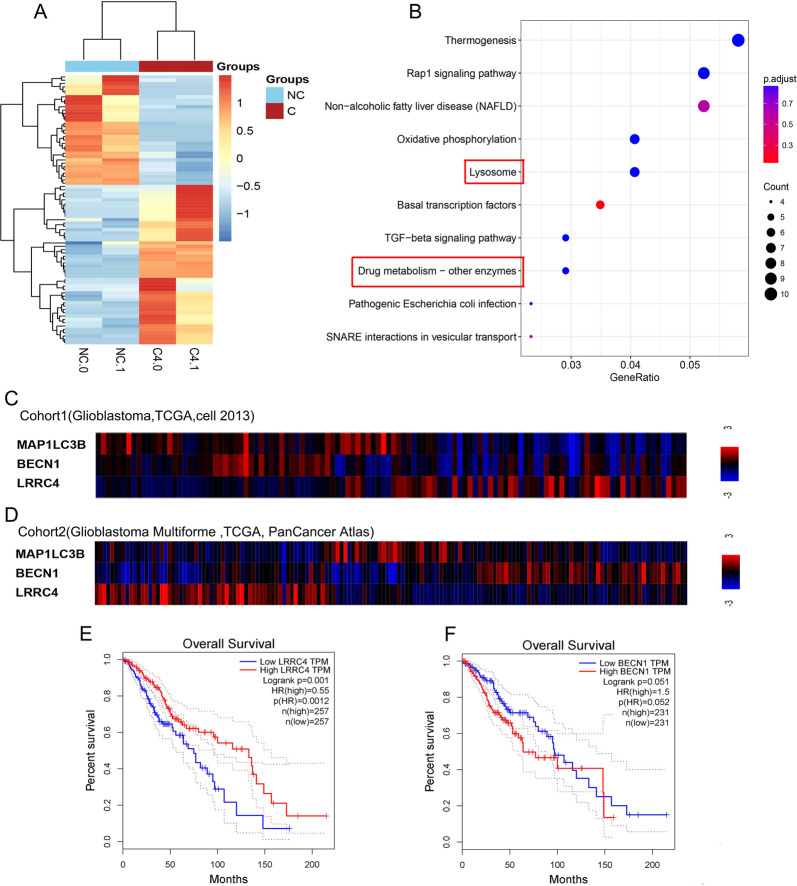
LRRC4 is negatively associated with autophagy signals and glioma patient outcomes. **a** Gene expression clustering diagram between U251 cells (NC: control) and U251 cells that stably expressed LRRC4 (C4: LRRC4). **b** KEGG pathway enrichment using differentially expressed genes between U251 cells (NC: control) and U251 cells that stably expressed LRRC4 (C4: LRRC4). The gene ratio means the proportion of genes related to this signalling pathway in the differentially expressed genes. Correlation analysis of LRRC4 and autophagy-related genes MAP1LC3Band BECN1 in Cohort 1 (Glioblastoma, TCGA, Cell 2013) (**c**) and Cohort 2 (Glioblastoma Multiforme, TCGA, PanCancer Atlas) (**d**) of GBM samples from TCGA database. Kaplan–Meier curve depicting survival of patients with low and high LRRC4 (**e**), BECN1 (**f**) in TCGA.

Our group have confirmed LRRC4 as a tumour suppressor for glioma by inhibiting GBM cell proliferation and invasion [24–26]. To investigate the new function of LRRC4 in glioma, we used RNA sequencing to analyse the differences in gene expression between LRRC4 stable ectopic expression and control U251 cell lines (Fig. 1a). Cluster profiler package was used to analyze these data, and result from KEGG pathway analysis showed that the differentially regulated genes participate in lysosomal function and drug metabolism (Fig. 1b), suggesting that LRRC4 may be associated with autophagy signalling and drug resistance in glioma. As autophagy plays an important role in drug resistance, we next focus our attention on the correlation between LRRC4 and autophagy pathway. We used the cBioPortal (http://www.cbioportal.org/) to analyse the correlations between LRRC4 and autophagy-related genes in GBM samples from TCGA database. In Cohort 1 (GBM, TCGA, Cell 2013), LRRC4 was negatively correlated with BECN1 and MAP1LC3B (Fig. 1c), which are genes that contribute to the origination of autolysosome and autophagy maker gene. Consistently, LRRC4 was also negatively correlated with BECN1 and MAP1LC3B in Cohort 2 (GBM Multiforme, TCGA, PanCancer Atlas) (Fig. 1d). We next evaluated the relationship between expression of LRRC4 and glioma survival rate. By using GEPIA (http://gepia.cancer-pku.cn/index.html), we found that the high expression LRRC4 group was strongly associated with longer survival (Fig. 1e), while BECN1 were seemingly associated with shorter survival (*p* = 0.051) (Fig. 1f). It suggested that glioma patients with high autophagy level have a worse prognosis than those patients with low autophagy level. Taken together, these data indicated that LRRC4 was inversely associated with autophagy signalling and glioma patient outcomes.

### LRRC4 inhibits autophagy activation in glioblastoma cells

**Fig. 2:**
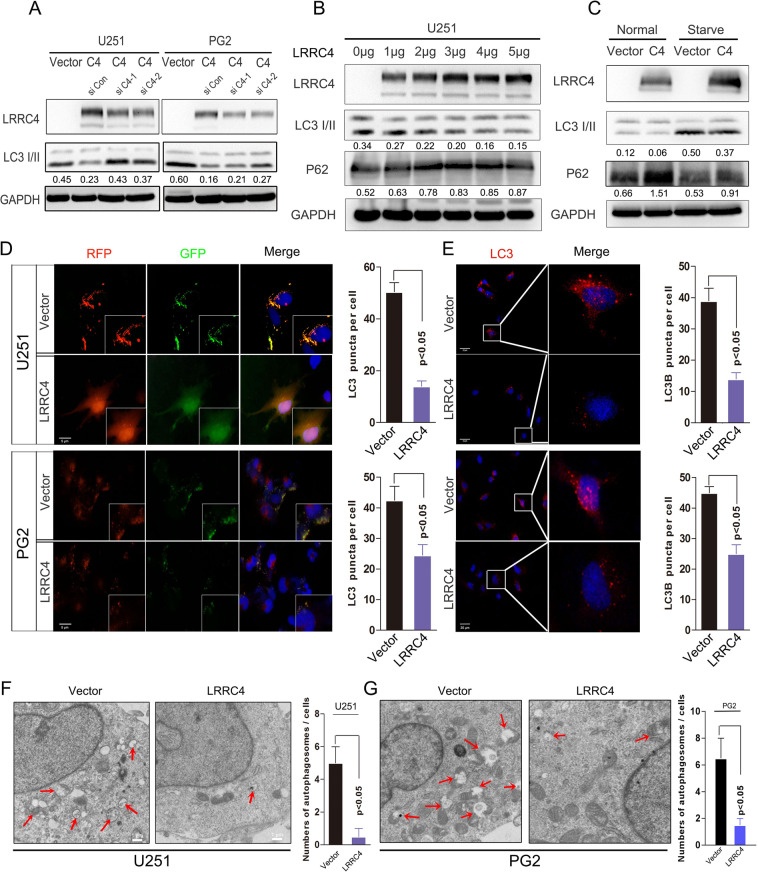
LRRC4 inhibits GBM cell autophagy activation. **a** The stably expressed LRRC4 decreased the expression of LC3B in U251 and PG2 cells. Knockdown LRRC4 restored the expression of LC3B in cells that stably expressed LRRC4. Cells were lysed and protein was extracted. LRRC4, LC3B and GAPDH were evaluated by western blotting. Vector: control plasmid, C4: LRRC4 plasmid. siC4-1 and siC4-2 are siRNAs that targeted LRRC4. Numbers represent the relative ratio of LC3II and GAPDH. **b** U251 cells were transfected with increasing concentrations of LRRC4 plasmid. Cells were lysed and protein was extracted. LRRC4, LC3B, P62 and GAPDH were evaluated by western blotting. LRRC4: LRRC4 plasmid. Numbers represent the relative ratio of LC3II and GAPDH, P62 and GAPDH. **c** U251 cells were transfected with LRRC4 or control plasmid and cells were cultured in normal and starvation conditions. LRRC4, LC3B, P62 and GAPDH were evaluated by western blotting. Vector: control plasmid, C4: LRRC4 plasmid. Numbers represent the relative ratio of LC3II, P62 and GAPDH. **d** U251 cells (upper) and PG2 cells (bottom) that stably expressed mRFP-EGFP-LC3 fusion protein were transfected with LRRC4 plasmid. Confocal microscopic analysis is shown. Scale bar, 5 μm. Vector: control plasmid, C4: LRRC4 plasmid. Note: PG2 cells were primary cultured glioblastoma cells. **e** U251 cells (upper) and PG2 cells (bottom) were transfected with LRRC4 plasmid. Cells were immunostained with an LC3B antibody. Fluorescence microscope analysis is shown. Scale bar, 20 μm. Vector: control plasmid, C4: LRRC4 plasmid. **f**, **g** Autophagosomes were observed by transmission electron microscopy in U251 cells and PG2 cells that were transfected with LRRC4 plasmid or control vector. Scale bar, 1 μm. Vector: control plasmid, LRRC4: LRRC4 plasmid.

How does LRRC4 regulate autophagy? Next, we examined the role of LRRC4 in cells autophagy via some cell biology experiments. LRRC4 is primarily expressed in normal brain tissues whilst lacking in GBM cells and primary glioma cells (Fig. S1). So, we conducted ectopic expression of LRRC4 to proceed with further investigation. Overexpression of LRRC4 decreased the expression of LC3B, a autophagy maker protein, in U251 cells and PG2 cells (Fig. 2a). LRRC4 knockdown restored the inhibition effect of LRRC4 on autophagy maker protein LC3B in stable LRRC4-overexpressing U251 and PG2 cells (Fig. 2a). To confirm this finding, p3.1-LRRC4 plasmid was transiently transfected into U251 cells at increasing plasmid concentrations. The results showed that LC3II was decreased and p62, which binds ubiquitin and LC3 and is a selective substrate for autophagy, expression was increased in a plasmid concentration-dependent manner (Fig. 2b). Starvation activated cell autophagy has been well described, and we wondered whether the inhibition of autophagy induced by LRRC4 could be restored. We compared the effect of LRRC4 on autophagy genes under normal conditions and starvation conditions. We found that LRRC4 also inhibited GBM cell autophagy in the starvation state. The results showed that under both conditions, LRRC4 inhibited GBM cell autophagy (Fig. 2c). We established U251 cells and PG2 cells (primary cultured GBM cells) that stably expressed a tandem mRFP-EGFP-LC3 plasmid and found that LRRC4 prevented the autophagic flux in U251 cells and PG2 cells (Fig. 2d). Under starvation conditions, in line with these results, an immunostaining assay also showed that LRRC4 decreased the level of endogenous LC3II in U251 cells and PG2 cells (Fig. 2e). In addition, autophagosomes were evaluated. Transmission electron microscopy showed a decrease in the formation of autophagic vesicles in the LRRC4-overexpressing U251 and PG2 cells (Fig. 2f). Collectively, these data indicated that LRRC4 inhibits the autophagy pathway in GBM cells.

### LRRC4^−/−^ mice display relatively higher levels of autophagy than LRRC4^+/+^ mice

**Fig. 3:**
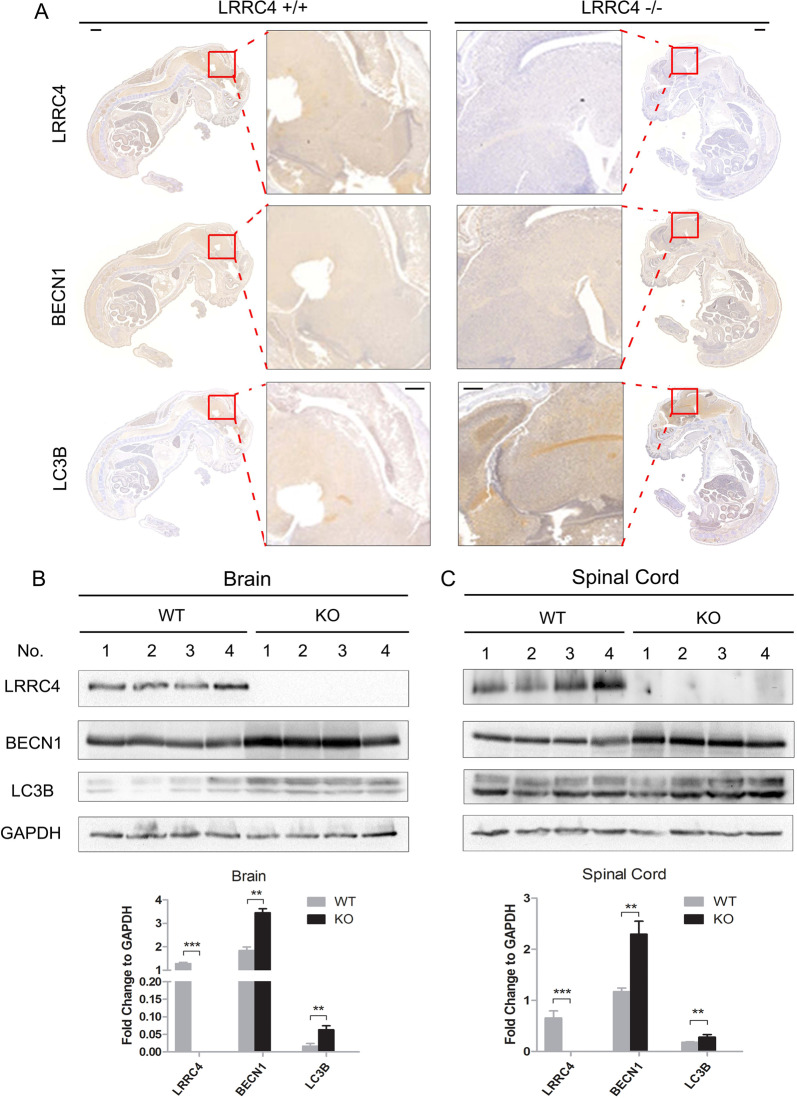
LRRC4^−^^/−^ mice display relatively higher levels of autophagy than LRRC4^+/+^ mice. **a** Representative whole-mount IHC analysis of LRRC4, BECN1 and LC3B for LRRC4 WT and KO mouse embryos at day 14.5 of gestation. Scale bar, 1 mm. Enlarged scale bar, 100 μm. Representative western blotting analysis of LRRC4, BECN1, LC3B and GAPDH in adult mouse brain (**b**) and spinal cords (**c**) from LRRC4 WT and KO mice. Statistical analysis is shown at the bottom.

To further validate the effect of LRRC4 on autophagy in vivo, we generated knockout (KO) mice that lacked exon 1 of the murine LRRC4 gene. Immunohistochemistry was used to analyse the expression of LRRC4, BECN1 and LC3B in E14.5 mouse embryos. Compared with LRRC4^+/+^ wild-type mice, the expression of BECN1 and LC3B was increased in LRRC4^−/−^ mice (Fig. 3a). Because LRRC4 is primarily expressed in the adult mouse nervous system, we next evaluated the expression of BECN1 and LC3B in the LRRC4^−^^/−^ adult mouse nervous system. As shown in Fig. 3a, c in both the mouse brain and spinal cord, the expression of BECN1 and LC3B was higher in LRRC4^−/−^ mice than in LRRC4^+/+^ mice. These data demonstrated that LRRC4 plays an important role in regulating autophagy signals under physiological conditions in mice. The above results also demonstrated that LRRC4 inhibits cell autophagy both in physiological and pathological conditions.

### LRRC4 restores GBM cell sensitivity to TMZ via the autophagy pathway

**Fig. 4:**
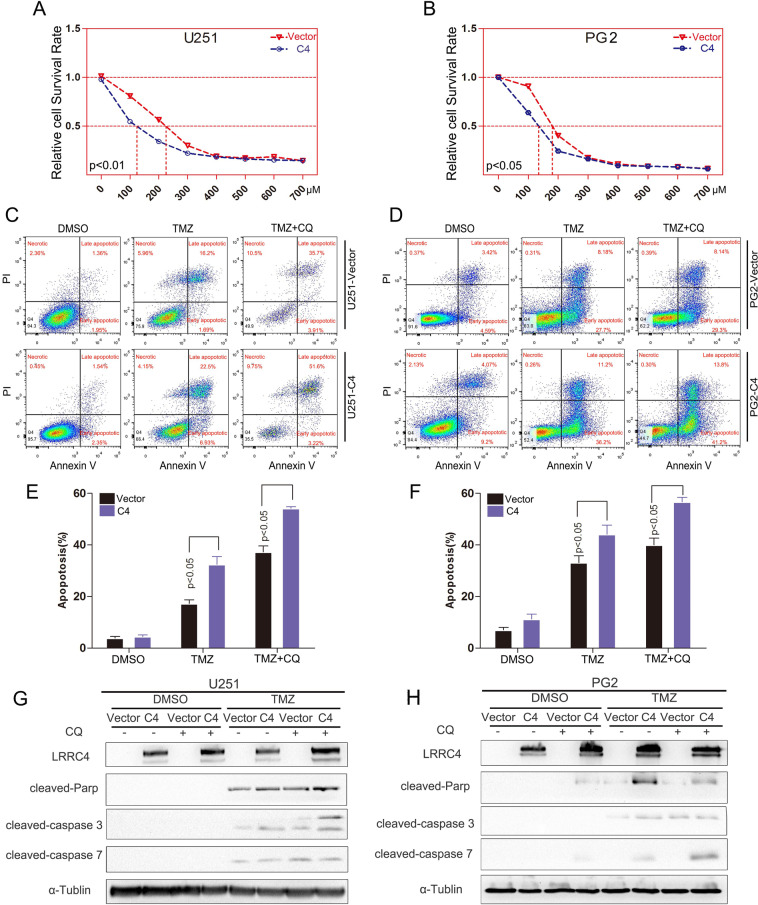
LRRC4 restores GBM cell sensitivity to TMZ. **a**, **b** U251 cells (left) and PG2 cells (right) that stably expressed control vector or LRRC4 (C4) were treated with different concentrations of TMZ. Cell viability was evaluated by CCK-8. **c**, **d** Apoptosis was detected by flow cytometry in U251 cells (left) and PG2 cells (right) that stably expressed control vector or LRRC4 (C4). The cells were treated with CQ (chloroquine) and TMZ (temozolomide). The images are representative of three independent experiments. **e**, **f** Statistical analysis was performed to calculate the apoptosis rate in **c** and **d**. **g**, **h** Cleaved caspase 3, 7 and parp expression was detected by western blotting in U251 cells (left) and PG2 cells (right). The cells were treated with CQ and TMZ. Vector: control plasmid, C4: LRRC4 plasmid.

It is well known that autophagy activation contribute to drug resistance. Thus, we hypothesized that LRRC4 could decrease GBM chemoresistance by inhibiting autophagy. To initially determine whether LRRC4 decreases GBM chemoresistance, LRRC4 stably expressing U251 and PG2 cells were treated with different TMZ concentrations, with doses ranging between 0 and 700 μM. We observed that the half inhibitory concentration (IC50) of TMZ in U251 cells with LRRC4 expression was ~122.3 μM; however, in control U251 cells, the IC50 was ~221.5 μM (Fig. 4a). The IC50 of TMZ was ~138.7 μM in PG2 cells with LRRC4 expression, whereas in the control PG2 cells, the IC50 was ~181.2 μM (Fig. 4b). These results showed that TMZ sensitivity was restored upon LRRC4 was expression in GBM cells. To further examine this hypothesis, cell apoptosis was examined by flow cytometry. As expected, TMZ induced U251 cell and PG2 cell apoptosis, and apoptosis increased in LRRC4-expressing U251 cells and PG2 cells compared with control cells (Fig. 4c, d). To address whether LRRC4 modulates the TMZ sensitivity of GBM cells via the autophagy pathway, we treated these cells with TMZ in the presence of chloroquine (CQ) or siRNA-mediated ATG5 and ATG7 gene knockdown. We found that the LRRC4-regulated TMZ-sensitivity effect was increased by CQ treatment (Fig. 4e, f) or knockdown of ATG5 or ATG7 in U251 cells and PG2 cells (Fig. S2). Moreover, in the presence of CQ, western blotting also showed that TMZ induced the cleavage of caspase 3, caspase 7 and PARP, and these effects were enhanced by LRRC4 expression in U251 cells and PG2 cells (Fig. 4g, h). Thus, these data suggest that LRRC4 restores TMZ-sensitivity in GBM by inhibiting autophagy and promoting apoptosis.

### LRRC4 combined with TMZ treatment prolonged mouse survival

**Fig. 5:**
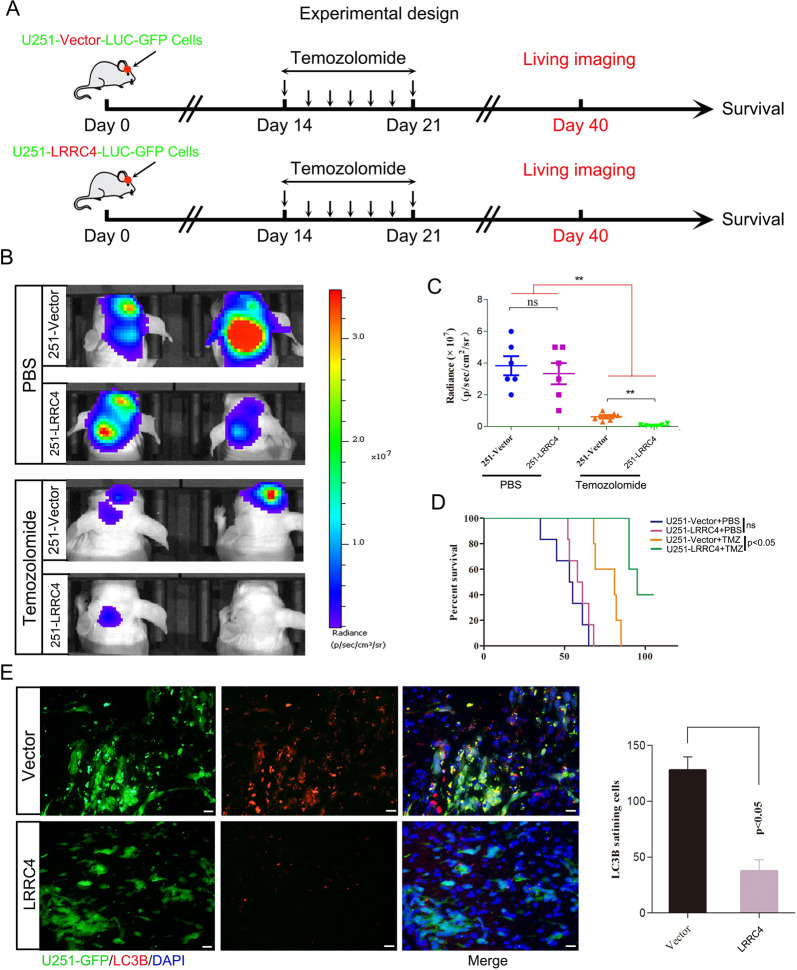
LRRC4 combined with TMZ treatment prolonged mouse survival. **a** Experimental design schematic showing the in vivo mouse model. **b** Representative bioluminescence images of mice intracranially injected with luciferase-labelled U251 cells that stably expressed vector or LRRC4 in the presence or absence TMZ treatment (*n* = 6 mice per group). **c** Quantitation of the fluorescence intensity of a representative image (*n* = 6 mice per group). **p < 0.01; **d** Kaplan–Meier survival curves of mice injected with U251 cells that stably expressed vector or LRRC4 in the presence or absence TMZ treatment (*n* = 6 mice per group). **e** Mouse brain sections stained for LC3B after intracranial transplantation of U251 cells that stably expressed vector or LRRC4. The right panel shows the quantitation of LC3B-positive cells.

The profound proapoptotic effects of the combination of LRRC4 expression and TMZ treatment in vitro suggest that targeting autophagic signalling may be a rational strategy for the treatment of GBM. To explore the feasibility of this therapeutic strategy, we tested the efficiency in tumour xenografts in vivo. U251-Luci-GFP-Vector or U251-Luci-GFP-LRRC4 cells were implanted into the brain of nude mice, and 14 days later, mice received intraperitoneal injections of TMZ (40 mg/kg/d) or saline once a day for 7 days (Fig. 5a). Imaging using an IVIS Lumina III In Vivo Imaging System showed no significant difference in the fluorescence signal in mice engrafted with U251-Luci-GFP-LRRC4 cells in comparison with that of animals implanted with U251-Luci-GFP-Vector cells (Fig. 5b, *p* = 0.0529). Nonetheless, when treated with TMZ, mice engrafted with U251-Luci-GFP-LRRC4 cells showed a significant decrease in fluorescence signal compared with that of mice implanted with U251-Luci-GFP-Vector cells (Fig. 5b). The relative fluorescence quantification is shown in Fig. 5c. Moreover, survival analysis showed that when mice that were intracranially engrafted with U251-Luci-GFP-LRRC4 cells were treated with TMZ, survival was longer than in animals implanted with U251-Luci-GFP-Vector cells (Fig. 5d) (*p* < 0.05). Immunofluorescence was used to confirm whether the decreased tumour growth observed in mice with LRRC4 overexpression combined with TMZ treatment was associated with an autophagy defect. Immunofluorescence staining in mouse brain sections demonstrated that LRRC4 expression inhibited LC3B expression in vivo (Fig. 5e), suggesting an autophagy deficiency in LRRC4-expressing cells. These results further confirmed that LRRC4 restores TMZ-sensitivity in GBM by inhibiting cell autophagy.

### LRRC4 binds to DEPTOR via the C-terminal PDZ binding domain

To investigate the mechanism by which LRRC4 inhibited cell autophagy, we used co-immunoprecipitation (CoIP) combined with mass spectrometry to identify its interaction partners, and DEPTOR (DEP domain containing MTOR interacting protein) was identified as a potential LRRC4 interaction partner (Fig. S3). DEPTOR interacts with both mTORC1 and mTORC2, and inhibits the activity of both mTORC1 and mTORC2 [29]. It is recognized that mTOR regulate autophagy through direct phosphorylation of Ulk1 [30]. So we then decided to concentrate on the DEPTOR.

**Fig. 6:**
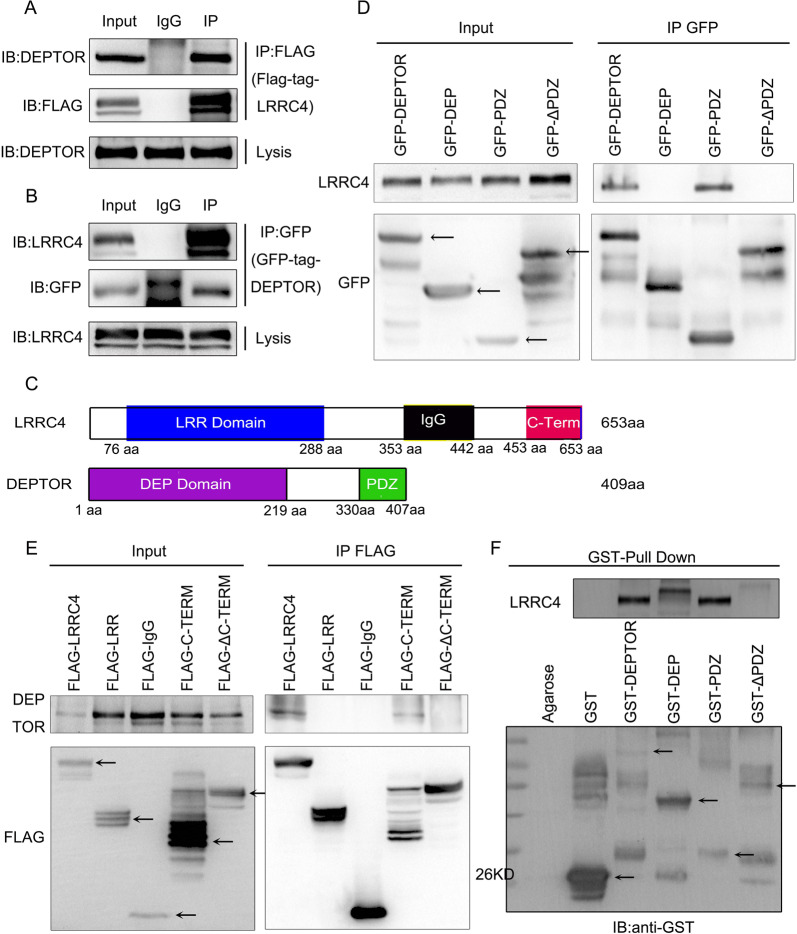
LRRC4 interacted with DEPTOR. **a** U251 cells were transfected with FLAG-LRRC4. Co-immunoprecipitation showed the interaction between LRRC4 and endogenous DEPTOR in U251 cells. **b** U251 cells were co-transfected with EGFP-C1-DEPTOR and pCDNA-3.1-LRRC4 plasmid. Co-immunoprecipitation showed the interaction between LRRC4 and DEPTOR in U251 cells. **c** Schematic diagrams of LRRC4 (upper)/DEPTOR (bottom) protein domains. **d** HEK293 cells were co-transfected with different GFP-tagged domains of DEPTOR and the pCDNA3.1-LRRC4 plasmid. Co-immunoprecipitation showed the interaction between LRRC4 and the PDZ domain of DEPTOR. **e** HEK293 cells were co-transfected with different FLAG-tagged domains of LRRC4 and pCDNA3.1-DEPTOR. Co-immunoprecipitation showed the interaction between DEPTOR and the C-terminal domain of LRRC4. **f** GST pull-down assays showed that the PDZ domain of DEPTOR pulled down LRRC4.

To test whether LRRC4 interacted with endogenous DEPTOR, a FLAG-tagged LRRC4 protein expression vector was transfected into HEK293 cells. The endogenous DEPTOR was co-immunoprecipitated with LRRC4 from the cell extract (Fig. 6a). Consistently, endogenous LRRC4 could also co-immunoprecipitate with GFP-tagged DEPTOR from the cell extract (Fig. 6b). To identify the interacting regions, we subsequently constructed plasmids containing different domains of LRRC4 and DEPTOR to perform CoIP assays. PDZ(330-407), containing the PDZ domain of DEPTOR, but not DEP (1-329), containing the DEP domain of DEPTOR, interacted with LRRC4, and deletion of the PDZ domain abolished the interaction between LRRC4 and DEPTOR (Fig. 6d). LRRC4 (443-653), containing the PDZ binding domain, but not other LRRC4 regions interacted with DEPTOR, and deletion of the C-terminal PDZ binding domain also abolished the interaction between LRRC4 and DEPTOR (Fig. 6e). In order to determine direct protein–protein interaction between DEPTOR and LRRC4, we subsequently performed a GST pull-down assay with LRRC4 and GST-fused DEPTOR full-length or different domains of DEPTOR (DEP: DEP domain, PDZ: PDZ domain or ΔPDZ: detection of PDZ domain in DEPTOR protein) as shown in Fig. 6c. The results revealed that the LRRC4 was pulled down by either the GST-fused full-length DEPTOR or GST-fused PDZ domain of DEPTOR, whereas the GST-fused DEP or ΔPDZ was not able to pull-down LRRC4 (Fig. 6f). Collectively, these data demonstrated that LRRC4 binds to DEPTOR directly through the C-terminal PDZ binding domain of LRRC4 and the PDZ domain of DEPTOR.

### LRRC4 promoted the degradation of the DEPTOR protein and re-expression of DEPTOR restored autophagy activation

**Fig. 7:**
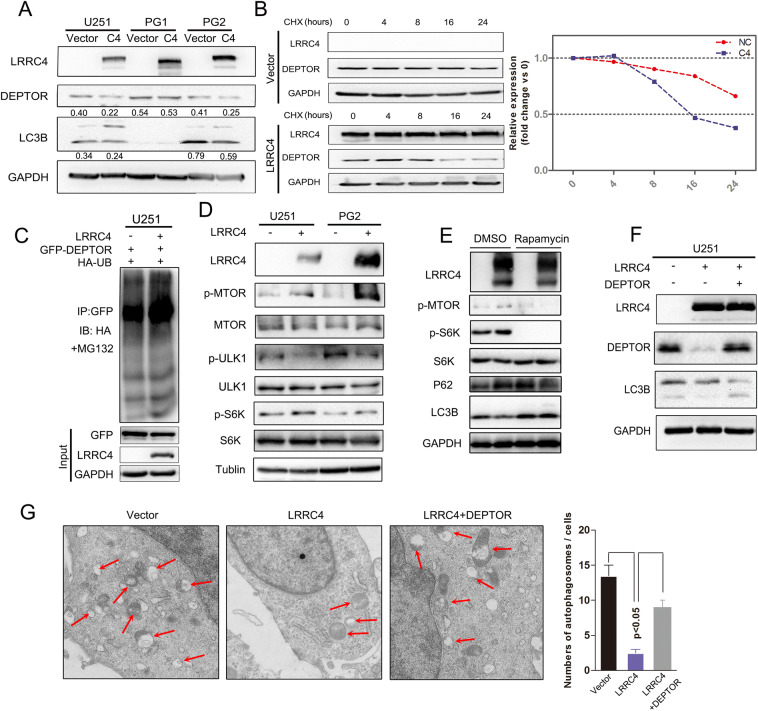
LRRC4 promoted the degradation of DEPTOR protein, and re-expression of DEPTOR restored autophagy activation. **a** Representative western blotting analysis of LRRC4, DEPTOR, LC3B and GAPDH in U251, PG1 and PG2 cells with or without transfection with LRRC4. Numbers represent the relative ratio of DEPTOR/LC3B and GAPDH. **b** Western blotting analysis was used to measure of the half-life of DEPTOR after treatment with cycloheximide in U251 cells with (down) or without (upper) LRRC4 transfection. Cells were lysed and protein was extracted at 0, 4, 8, 16, 24 h after cycloheximide treatment. **c** DEPTOR ubiquitination was assessed by an anti-GFP antibody in the presence of MG132 when GFP-DEPTOR, HA-ubiquitin, and LRRC4 or vector were co-transfected into U251 cells. **d** Representative western blotting analysis of LRRC4, p-MTOR, MTOR, p-ULK1, ULK1, p-S6K, S6K and Tubulin in U251 and PG2 cells with or without transfection with LRRC4. **e** Representative western blotting analysis of LRRC4, p-MTOR, p-S6K, S6K, P62, LC3B and GAPDH in U251 cells, U251 cells with or without LRRC4 transfection were treated with dimethylsulfoxide (DMSO) or Rapamycin. **f** Representative western blotting analysis of LRRC4, DEPTOR, LC3B and GAPDH in U251 cells, U251 cells with stable expression LRRC4 and LRRC4-expressing U251 cells with ectopic expression of DEPTOR. **g** Autophagosomes were observed by transmission electron microscopy in U251 cells, U251 cells with stable expression LRRC4 and LRRC4-expressing U251 cells with ectopic expression of DEPTOR.

DEPTOR is an unstable protein and can be degraded under growth factor stimulation [29]. Previous studies also reported that DEPTOR was degraded by SCF^βTrCP^ [30]. Next, we wanted to determine whether the interaction between LRRC4 and DEPTOR could alter the expression of DEPTOR. We found that DEPTOR expression was decreased in U251 and PG2 cells that were transfected with an LRRC4 plasmid while not in PG1 cells (Fig. 7a). In line with that the overexpression of LRRC4 in PG1 has no effect on autophagy inhibition. It suggested that LRRC4 inhibited autophagy via downregulating DEPTOR. We next examined whether LRRC4 affected the stability of DEPTOR using a cycloheximide (CHX)-chase assay. The half-life of DEPTOR was decreased by overexpression of LRRC4 in U251 cells (Fig. 7b). In addition, we also examined the effect of overexpressing LRRC4 on DEPTOR ubiquitin modifications, and the ubiquitin modifications of DEPTOR were also increased by transfected LRRC4 (Fig. 7c).

To further demonstrate the effects of regulation by LRRC4 on DEPTOR expression and autophagy, U251 and PG2 cells were transiently transfected with LRRC4 plasmid. The overexpression of LRRC4 could induce mTOR activation, as shown by increased expression of phospho-MTOR and phospho-S6K (Fig. 7d), which was consistent with the precious conclusion that the cell autophagy level could be inhibited by MTOR pathway activation. Furthermore, when GBM cells treated with pharmacological MTOR inhibitors (Rapamycin), the inhibition effect of LRRC4 on autophagy was broke, as the difference of p62 and LC3B protein level between vector and LRRC4-expressing group had no significant changes (Fig. 7e). Moreover, re-expression of DEPTOR rescued cell autophagy in LRRC4 stable expression U251 cells, as Fig. 7f shows increased protein levels of LC3B. Similarly, re-expression of DEPTOR could restore the formation of autophagic vesicles in LRRC4-expressing U251 cells (Fig. 7g). Together, these results demonstrate that LRRC4 inhibits cell autophagy by promoting DEPTOR degradation and that re-expression of DEPTOR blocks this suppressive effect.

### The levels of LRRC4, DEPTOR and LC3B are clinically relevant for GBM

**Fig. 8:**
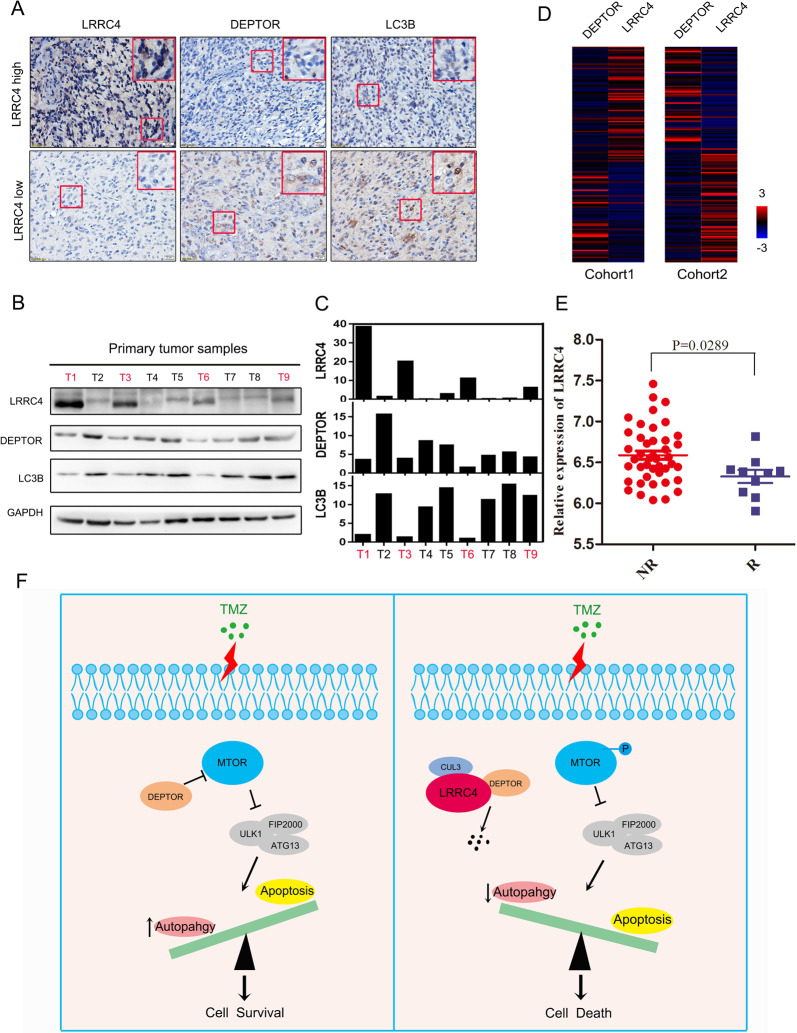
The Levels of LRRC4, DEPTOR and LC3B are clinically relevant for GBM. **a** Representative immunohistochemistry staining of LRRC4, DEPTOR and LC3B proteins in GBM tissues. (LRRC4 high GBM tissues = 5, LRRC4 low GBM tissues = 27). **b** Representative western blotting analysis of LRRC4, DEPTOR and LC3B proteins in primary GBM tissues. LRRC4 is negatively correlation with DEPTOR and LC3B expression. T tumour. **c** The quantitative analysis of LRRC4, DEPTOR and LC3B proteins expression in each primary GBM tissues (**b**). **d** The correlation analysis of LRRC4 and DEPTOR in Cohort 1 (Glioblastoma, TCGA, Cell 2013, left) and Cohort 2 (Glioblastoma Multiforme, TCGA, PanCancer Atlas, right) of GBM samples from TCGA database. **e** Statistical analysis of the expression of LRRC4 between recurrent and non-recurrent patients from the Oncomine Murat brain dataset. **f** Schematic diagram of the relationship among LRRC4, autophagy and TMZ chemosensitivity: when GBM cells without LRRC4 expression are attacked by TMZ treatment, DEPTOR directly binds and inhibits the phosphorylation of MTOR, thereby trigger protective autophagy to permit GBM cell survival. But, when GBM cells with LRRC4 expression are attacked by TMZ treatment, LRRC4 directly binds and promotes the degradation of DEPTOR. Degradation of DEPTOR leads to activation of MTOR, thereby inhibits protective autophagy, and finally induces apoptotic cell death.

To investigate the clinical significance of LRRC4, DEPTOR and LC3B in GBM, we detected the protein expression of LRRC4, DEPTOR and LC3B by immunohistochemistry in GBM tissues. We found that high expression of LRRC4 was more likely to be detected in patients with low expression of DEPTOR and LC3B. In contrast, low expression of LRRC4 was more likely detected in patients with high expression of DEPTOR and LC3B (Fig. 8a). Furthermore, we examined the LRRC4, LC3B and DEPTOR protein expression in total protein extraction of GBM specimens. As showed in Fig. 8b, DEPTOR was expressed in most GBM tissues, while in LRRC4 expressed tissues (T1, T3, T6 and T9), the expression of DEPTOR and LC3B protein was relative low compared with no-LRRC4 expression tissues (Fig. 8c).

We also studied the relationship of LRRC4 and DEPTOR in Cohort 1 and Cohort 2, as mentioned in Fig. 1. Similarly, LRRC4 was also negatively correlated with DEPTOR in Cohort 1 and Cohort 2 (Fig. 8d). As drug resistance contribute to tumour recurrence, we also evaluated the expression of LRRC4 mRNA in GBM and recurrent GBM patients by using the Oncomine Murat brain dataset. As shown in Fig. 8e, reduced LRRC4 mRNA levels were found in recurrent GBM samples compared with GBM samples without recurrence, suggesting that the expression levels of LRRC4 may be negatively correlated with drug resistance. Altogether, we conclude that LRRC4 may act in GBM via DEPTOR, causing MTOR pathway activation, which subsequently results in autophagy inhibition, and consequently promotes TMZ chemosensitivity in GBM patients (Fig. 8f).

## Discussions

The LRRC4 gene was first identified on human chromosome 7q31-32 by our group [19], and our reports confirmed that LRRC4 is a tumour suppressor gene for glioma [27, 28]. Although the function of LRRC4 has been reported in our previous work, it is unknown whether LRRC4 may mediate autophagy in glioma. We found that expression of LRRC4 inhibited autophagic flux and autophagosome synthesis in GBM cells. Accordingly, LRRC4 decreased the expression of the autophagy-related proteins LC3B in GBM cells, suggesting that LRRC4 prevents autophagy pathway activation. We also examined the relationship of LRRC4 and autophagy in LRRC4 gene KO mice and found that the lrrc4^−/−^ mice display relatively high levels of autophagy-related proteins compared with those of WT mice in mouse brain and spinal cord. Previous studies have shown that LRRC4 regulates auditory responses [31], excitatory synapse development, synapse maintenance and restoration in the retina [32] and autistic-like behaviours that are responsive to NMDAR modulation [33] by using *lrrc4* KO mice. Our unpublished results showed that lrrc4^−^^/−^ mice display a more serious phenotype than WT mice in the EAE model, and re-expressing LRRC4 in lrrc4^−/−^ mice could partially rescue the phenotype. Autophagy dysfunction in neurodegenerative disorders has been widely reported [34, 35]. Our study reveals that LRRC4 regulates autophagy in the mouse nervous system. This may explain why LRRC4 dysfunction contributes to neurological function disorders in a mouse model.

TMZ, an FDA-approved chemotherapy drug, has been widely used to treat glioma [36]. Although glioma patients often initially respond to surgical resection and chemotherapy, relapse of drug-resistant cancer usually occurs, and treatment is usually ineffective [37]. Unfortunately, due to the existence of the blood–brain barrier, potentially powerful anticancer drugs and novel immune checkpoint therapy are ineffective for GBM [38]. TMZ remains a first-line therapy for patients with GBM. Thus, understanding the mechanisms of TMZ resistance in GBM or exploring prognostic markers that predict TMZ chemosensitivityis essential to optimize current therapeutic strategies. It has been reported that chemotherapy can induce autophagy activation in tumour cells, and some articles have also discussed a strategy that targets autophagy to sensitive glioma to TMZ treatment [39–41]. Our study found that TMZ treatment induced the expression of autophagy-related proteins BECN1 and LC3B (data not shown). Hence, we hypothesized that LRRC4 expression could promote the sensitivity of GBM to TMZ treatment. We confirmed that LRRC4 induced GBM cell apoptosis when treated with TMZ, and the combination of biochemical autophagy inhibition (CQ) with LRRC4 expression significantly enhanced the cell apoptosis rate. Thus, we conclude that autophagy contributes to LRRC4-mediated GBM responses to TMZ regimens. These results support the phenomenon that GBM patients with low expression of LRRC4 experience poor outcomes and low TMZ chemosensitivity.

We have described the mechanisms by which LRRC4 inhibits autophagy pathway activation. DEPTOR was found to interact with LRRC4 by MS analysis. DEPTOR is a naturally occurring inhibitor of mTOR that directly binds to both mTORC1 and mTORC2 [29]. DEPTOR is subject to proteasome-dependent degradation [30], and the degradation of DEPTOR contributes to mTOR activation, thus inhibiting the cell autophagy pathway [42]. Our data showed that LRRC4 induces the degradation of DEPTOR by directly interacting with DEPTOR. We also confirmed that overexpression of LRRC4 induced phosphorylation of mTOR and S6K1, which was accompanied by decreased expression of the autophagy-related proteins LC3B. This result supports the conclusion that LRRC4 inhibits GBM cell autophagy via the degradation of DEPTOR. DEPTOR acts as a tumour suppressor by blocking mTORC1 and mTORC2, inhibiting cell proliferation. However, studies have also demonstrated that DEPTOR is overexpressed in many tumours, including breast, prostate and lung cancers [43–45], indicating that DEPTOR also acts as an oncogene during tumour growth. DEPTOR overexpression is able to inhibit mTORC1, leading to an apparent increase in mTORC2 signalling, inducing Akt phosphorylation at S437 and T308 residues [46]. Efeyan found that DEPTOR could relieve the feedback inhibition from S6K1 to PI3K, thus activating AKT [47]. Wang also reported that DEPTOR was a novel target of Wnt/b-Catenin/c-Myc and contributed to colorectal cancer cell growth [48]. This may explain why LRRC4 expression leads to mTOR activation but does not contribute to cell proliferation.

In conclusion, our results demonstrate that LRRC4, which is frequently deregulated in glioma, directly binds to DEPTOR and induces its degradation to activate mTOR, thereby inhibiting cell autophagy. Moreover, autophagy inhibition increased the treatment efficacy of TMZ in glioma, and LRRC4-expressing cells underwent increased apoptosis with TMZ treatment. Importantly, in clinical glioma samples, LRRC4 was also negatively associated with DEPTOR and LC3 expression. Combined LRRC4 expression and TMZ treatment could be an effective strategy for glioma therapy. Thus, the expression of LRRC4 is likely to have significant potential as a therapeutic marker and target for TMZ treatment in glioma patients.

## Materials and methods

### Tissue samples

Primary glioma samples and normal brain tissue were obtained from the Department of Neurosurgery at Xiangya Hospital in Hunan, China. All of the protocols were reviewed by the Joint Ethics Committee of the Central South University Health Authority and performed following national guidelines. Primary tumour and normal brain tissue were frozen in liquid nitrogen and stored until total proteins were extracted.

### Primary cell culture

Primary cell (PG1 and PG2) culture protocols were described in our previous report [28].

### Cell culture and reagents

U251, HEK293, PG1 and PG2 cells were maintained in DMEM supplemented with 10% foetal bovine serum and antibiotics. Cells were incubated at 37 °C in a humidified atmosphere of 5% CO_2_. U251 cells were authenticated to have originated from ATCC by short tandem repeat profiling. For Starvation induced autophagy, GBM cells were transfected with LRRC4 plasmid or control vector, after 42 h, the cells were washed twice with D-hanks and were stimulated with EBSS (6 h) as amino-acid starvation conditions. Cell extracts were immunoblotted with the indicated antibodies. Antibodies against GAPDH (60004-1-Ig), GST (66001-1-Ig), HA (51064-2-AP) and P62 (18420-1-AP) were purchased from Proteintech. Antibodies against LRRC4 (ab116697) were purchased from Abcam. FLAG (F1804) was purchased from Sigma-Aldrich. Antibodies against Beclin-1 (D40C5), LC3B (D11), mTOR (7C10), phospho-mTOR (Ser2448) (D9C2), DEPTOR/DEPDC6 (D9F5) and an apoptosis antibody sampler kit (#9915) were purchased from Cell Signaling Technology.

### Immunoprecipitation and immunoblotting

The protocols were described in our previous reports. Briefly, for immunoprecipitation, cells were lysed in IP buffer (25 mM Tris pH 7.5, 150 mM NaCl, 1% Triton X-100 and 1 mM EDTA). Cell lysates were incubated with the indicated antibodies at 4 °C overnight. Protein A/G Magnetic Beads were washed twice with IP buffer and were then added to the reaction mixtures and incubated for 2 h at 4 °C. After magnetic separation, the magnetic beads were washed four times with IP buffer and boiled for 10 min after the addition of 2 × SDS loading buffer. The immunoprecipitated proteins were analysed by SDS-PAGE. For immunoblotting, cells were lysed in RIPA buffer (100 mM Tris pH 7.4, 150 mM NaCl, 5 mM EDTA, 1% Triton X-100, 1% deoxycholate acid, 0.1% SDS, 2 mM phenylmethylsulfonyl fluoride, 1 mM sodium orthovanadate, 2 mM DTT, 2 mM leupeptin, 2 mM pepstatin). Protein lysates were fractionated by SDS-polyacrylamide gel electrophoresis, transferred onto PVDF membranes (Merck Millipore, Germany), and then incubated with the indicated primary antibodies, washed and probed with HRP (Horseradish peroxidase)-conjugated secondary antibodies.

### Pull-down assay

The protocol was described in our previous reports. Briefly, glutathione-S-transferase (GST) fusion proteins containing various regions of DEPTOR were expressed in BL21 (DE3) bacteria with a pGEX-4T-2 vector and were purified. Various FLAG-tagged sections of LRRC4 were transfected into HEK293 cells and were lysed in IP buffer. Then, the lysate was incubated for 4 h with GST-tagged proteins and glutathione-Sepharose 4B beads. The beads were subsequently washed four times in IP buffer. Precipitates were separated by SDS-PAGE and detected by western blot analysis.

### Immunofluorescence

The cultured cells were plated on coverslips and transfected with the indicated plasmids. After 48 h, the cells were washed and fixed in 4% paraformaldehyde at room temperature for 30 min. The cells were then washed two times with 0.1% PBS-T. For permeabilization, cells were incubated with 0.25% Triton X-100 in PBS for 15 min and washed two times with 0.1% PBS-T. Cells were incubated in blocking solution (normal goat serum) for 30 min to block nonspecific binding of the antibody and were incubated in primary antibodies diluted in PBS. After four washes with 0.1% PBS-T, cells were incubated in secondary antibodies for 1 h at room temperature. Coverslips were mounted and imaged by fluorescence microscopy.

### Xenograft tumour model

All animal experiments were approved by the Animal Care and Use Committee of Central South University. U251-GFP-Luci-Vector or U251-GFP-Luci-LRRC4 cells were collected, resuspended at 5 × 10^5^ cells in 5 μl of serum-free medium per animal, and then stereotactically injected into the striatum (1.0 mm anterior and 1.0 mm lateral from the Bregma suture and 3.0 mm below the pial surface) of nude mice. A total of 24 mice were used for the intracranial xenograft tumour model, six mice per group. TMZ was formulated in 3% DMSO, 50% PEG300 and 0.5% Tween 80 as suggested by Selleck Chemicals. Cells were implanted into brain of nude mice, and 14 days later, mice received intraperitoneal injections of TMZ (40 mg/kg/d) or saline once a day for 7 days. Tumour growth was measured 45 days after tumour inoculation using an IVIS Lumina III In Vivo Imaging System.

### Immunohistochemistry

GBM tumour and mouse paraffin sections were dewaxed, rehydrated and antigen retrieval was performed. Sections were blocked with 3% hydrogen peroxide for 10 min and with normal goat serum for 30 min at room temperature. Then, the sections were incubated with anti-LRRC4 (Abcam), DEPTOR (Cell Signaling Technology), BECN1 (Proteintech) and LC3B (Cell Signaling Technology) antibodies for 12 h at 4 °C and were incubated with biotinylated secondary antibody (Maxim Biotechnologies) for 30 min at room temperature and streptavidin conjugated HRP for 30 min. Staining was visualized with 3,3-diaminobenzidine (DAB; Maxim Biotechnologies) and was counterstained with haematoxylin.

### Apoptosis measurement

Apoptosis was assessed by labelling cells with annexin-V-FITC and propidium iodide (PI). Cells were then analysed (*n* = 20,000) by flow cytometry (Accuri C6 cytometer, Becton-Dickinson). Annexin V-positive cells (PI negative and positive) were considered apoptotic.

### Electron microscopy

Cells were treated as indicated and fixed with 2.5% glutaraldehyde containing 0.1 mol/l sodium cacodylate. Samples were fixed using 1% osmium tetroxide, followed by dehydration with an increasing concentration gradient of ethanol and propylene oxide. Samples were then embedded, cut into 50-nm sections, and stained with 3% uranyl acetate and lead citrate. Images were acquired using a JEM-1200 electron microscope (JEOL).

### Statistical analysis

Data are presented as the mean ± SD from at least three separate experiments, and the data were analysed with GraphPad Prism 5 (La Jolla, CA, USA). Differences between variables of two groups were examined by Student’s *t* test, and one-way ANOVA was used to evaluate the differences among variables of multiple groups. OS curves for the xenograft tumour model mice were calculated by the Kaplan–Meier method. The results were considered significant when *p* < 0.05 was obtained.

## Supplementary information

Supplemental Figures and Supplemental Figure legends

Supplemental LRRC4 MS data

Supplemental NC_C_0X1_GENE expression_estimate

## Data Availability

All authors ensure that all data generated or analysed during this study are included in this published article.
