# Supplementary material for: Leucine-rich repeat containing 4 act as an autophagy inhibitor that restores sensitivity of glioblastoma to temozolomide
Source: Oncogene. 2020 May 5;39(23):4551–66. doi: 10.1038/s41388-020-1312-6 (PMC7269909; doi:10.1038/s41388-020-1312-6)
Supplement: Supplementary file 1 — Supplemental Figures and Supplemental Figure legends [file 41388_2020_1312_MOESM1_ESM.docx]

**Leucine-rich repeat containing 4 act as an autophagy inhibitor that restores sensitivity of glioblastoma to Temozolomide**

**Supplemental Figures and Supplemental Figure legends**

**Figure S1 LRRC4 expression in normal brain but not GBM cells**


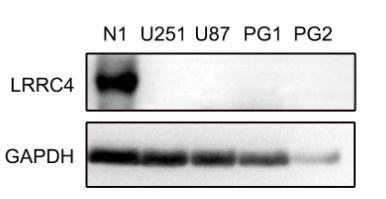


Western blotting showed LRRC4 expressed in in normal brain tissue (N1) while it lacking in GBM cells (U251 and U87) and primary glioma cells (PG1 and PG2)

**Figure S2 LRRC4-regulated TMZ-sensitivity effect was enhanced by ATG5 and ATG7 siRNAs transfection**


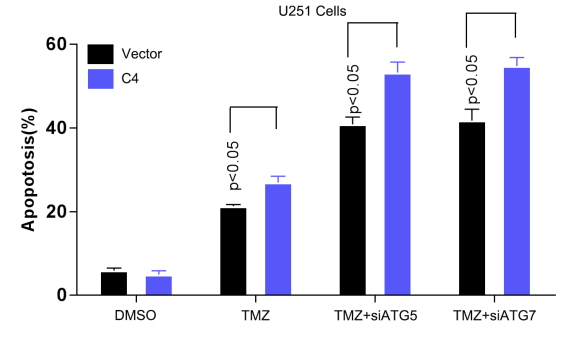

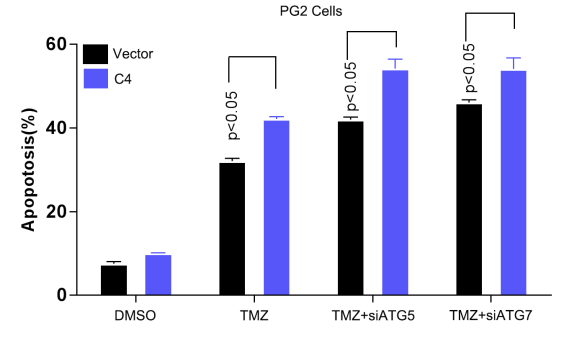


Flow cytometry showed that ATG5 and ATG7 siRNAs transfection enhanced TMZ induced apoptosis in the presence LRRC4 in U251 and PG2 cells.

**Figure S3 Silver staining and MS analysis the interactor of LRRC4**


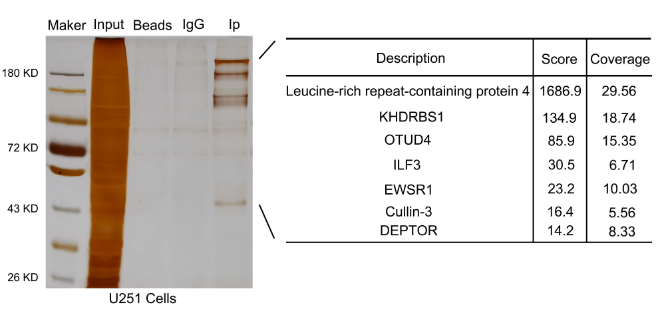


Silver staining (left) showed the Protein bands co-immunoprecipitated by flag-LRRC4 (line 5). Table showed some proteins from MS date.

**Figure S3 The relationship between LRRC4, DEPTOR and LC3B expression.**


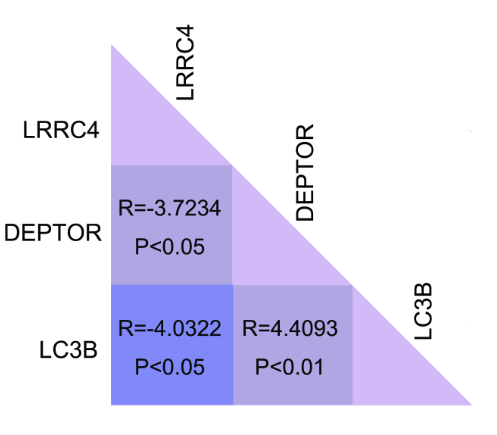


Statistical analysis of Figure 8A: We found positive correlation between the level of DEPTOR and that of LC3B, While LRRC4 levels correlated inversely with the levels of both DEPTOR and LC3B.
